# Dexmedetomidine versus remifentanil in postoperative pain control after spinal surgery: a randomized controlled study

**DOI:** 10.1186/s12871-015-0004-1

**Published:** 2015-02-24

**Authors:** Wonjung Hwang, Jaemin Lee, Jihyun Park, Jin Joo

**Affiliations:** Department of Anesthesiology and Pain Medicine, Seoul St. Mary’s Hospital, College of Medicine, The Catholic University of Korea, 222 Banpodaero, Seocho-gu, Seoul 137-701 South Korea

**Keywords:** Dexmedetomidine, Remifentanil, Total intravenous anesthesia, Postoperative pain

## Abstract

**Background:**

Total intravenous anesthesia (TIVA) is used widely in spinal surgery because inhalational anesthetics are known to decrease the amplitude of motor evoked potentials. Presently, dexmedetomidine is used as an adjuvant for propofol-based TIVA. We compared the effects of remifentanil and dexmedetomidine on pain intensity as well as the analgesic requirements after post-anesthesia care unit (PACU) discharge in patients undergoing spinal surgery.

**Methods:**

Forty patients scheduled for posterior lumbar interbody fusion (PLIF) surgery under general anesthesia were enrolled. Anesthesia was maintained using propofol at 3–12 mg/kg/h and remifentanil at 0.01–0.2 μg/kg/min in Remifentanil group or dexmedetomidine at 0.01–0.02 μg/kg/min in Dexmedetomidine group, keeping the bispectral index between 40 and 60. Patient-controlled analgesia (PCA) made of hydromophone was applied once the patients opened their eyes in the PACU. The visual analog scale (VAS) score, PCA dosage administered, and postoperative nausea and vomiting (PONV) were recorded at the time of discharge from the PACU (T1) and at 2 (T2), 8 (T3), 24 (T4), and 48 hours (T5) after surgery.

**Results:**

The VAS score in Remifentanil group was significantly higher than that in Dexmedetomidine group at immediate and late postoperative period (4.1 ± 2.0 vs. 2.3 ± 2.2 at T1, and 4.0 ± 2.2 vs. 2.6 ± 1.7 at T5; *P* < 0.05). Dexmedtomidine group had a statistically significantly lower PCA requirement at every time point after surgery except directly before discharge from the PACU (3.0 ± 1.2 ml vs. 2.3 ± 1.4 ml at T1; *P* > 0.05, but 69.7 ± 21.4 ml vs. 52.8 ± 10.8 ml at T5; *P* < 0.05). Patients in Remifentanil group displayed more PONV until 24 hours post-surgery.

**Conclusions:**

Dexmedetomidine displayed superior efficacy in alleviating pain and in postoperative pain management for 48 hours after PLIF. Therefore, dexmedetomidine may be used instead of remifentanil as an adjuvant in propofol-based TIVA.

**Trial registration:**

Clinical Research Information Service (CRiS) Identifier: KCT0001041.

## Background

Total intravenous anesthesia (TIVA) is widely used in spinal surgery because inhalational anesthetics are known to decrease the amplitude of motor evoked potentials, an important method of intraoperative monitoring [1,2]. Remifentanil is a standard adjuvant for propofol-based TIVA, having a rapid onset and ultra-short duration of action. Although remifentanil provides rapid recovery from anesthesia, long-term infusion may cause opioid-induced hyperalgesia (OIH) [3,4].

α2-adrenoreceptor agonist have been used as the sole analgesic agents during and after surgery [5]. Dexmedetomidine is a selective α2-adrenoreceptor agonist possessing properties of sedation, anxiolysis, and analgesia without the development of respiratory depression [6,7]. Its shorter duration of action (plasma half-life ~2.3 hours) comparing to clonidine and anesthetic-sparing effect have led to dexmedetomidine usage as an adjuvant in general anesthesia [8-10]. In relation to this, dexmedetomidine is now used increasingly as an adjuvant for propofol-based TIVA [10,11].

Several studies have shown that dexmedetomidine has superior efficacy compared to remifentanil and other opioids in immediate postoperative pain management in the post-anesthesia care unit (PACU) [8,12,13]. However, no studies have reported whether dexmedetomidine or remifentanil as an adjuvant in propofol-based TIVA results in differences in long-term postoperative pain and recovery quality after discharge from the PACU. Therefore, we compared the effects of remifentanil and dexmedetomidine on pain intensity, analgesic requirements, and postoperative nausea and vomiting (PONV) after discharge from the PACU in patients undergoing spinal surgery.

## Methods

This study was approved by the Ethical Committee of Seoul St. Mary’s Hospital, Catholic University of Korea, and was registered at Clinical Research Information Service (CRiS, http://cris.nih.go.kr, ID: KCT0001041). We obtained written informed consent from the participants. Forty patients (aged 18–70 years, American Society of Anesthesiologists physical status I or II) who were suffering from lumbar herniated nucleus pulposus, spinal stenosis, spondylolysis and spondylolisthesis, and scheduled for posterior lumbar interbody fusion (PLIF) surgery under general anesthesia were enrolled from September 2013 to January 2014. Patients with coronary artery or ischemic disease, who had bradycardia (<50 bpm) or an arrhythmia, or who were allergic to the study drugs were excluded. The patients were allocated to Remifentanil group or Dexmedetomidine group, receiving remifentanil or dexmedetomidine, respectively, as a TIVA adjuvant using computerized single block randomization. The drugs were prepared in a 50-ml syringe mixed with normal saline.

The patients were not premedicated, and a 20-gauge venous cannula was inserted to administer Ringer’s lactated solution. On arrival in the operating room, noninvasive blood pressure monitoring, electrocardiography using lead II, pulse oximetry, and capnography were applied and performed continuously. Bispectral index (BIS) electrodes were placed on the forehead to monitor the degree of anesthesia. Prior to anesthesia induction in Remifentanil group, 0.01 μg/ kg/min of remifentanil (i.e., 0.5 μg/min for 50 kg patient) was administered continuously using target-controlled infusion (TCI) (Orchestra® Workstation; Fresenius Kabi, Bad Homburg, Germany), whereas 0.01 μg/kg/min of dexmedetomidine (i.e., 0.5 μg/min for 50 kg patient) was administered continuously using a syringe pump (Terufusion® Syringe Pump; Terumo Corp., Tokyo, Japan) in Dexmedetomidine group. After 10 minutes of study drug infusion, 1–2 mg/kg of propofol was manually administered in increments of 20 mg every 15 seconds until BIS reached 40–50. When the patients were fully sedated (BIS 40–50), 1 mg/kg of rocuronium was administered and the trachea was intubated after manual ventilation for 1 minute. Anesthesia was maintained using propofol at 3–12 mg/kg/h (i.e., 150–600 mg/hr for 50 kg patient) using TCI (Orchestra® Workstation; Fresenius Kabi) with remifentanil at 0.01-0.2 μg/kg/min (i.e., 0.5-10 μg/min for 50 kg patient) in Remifentnai group or dexmedetomidine at 0.01–0.02 μg/kg/min (e.g. 0.5-1.0 μg/min for 50 kg patient) in Dexmedetomidine group, keeping the BIS between 40 and 60 and hemodynamic changes < 20% of baseline in both groups. Mechanical ventilation was maintained using air (50%) and oxygen (50%), with an end-tidal CO_2_ of 30–40 mmHg in both groups. Remifentanil was discontinued on completion of skin closure in Remifentanil group, whereas dexmedetomidine was ceased when skin closure was started in Dexmedetomidine group, taking into consideration their respective half-times [14,15]. Propofol was terminated upon the completion of skin closure.

On completion of surgery, 0.3 mg of ramosetron was administered for PONV, while 0.2 mg/kg of pyridostigmine and 0.008 mg/kg of glycopyrrolate were administered to reverse muscle relaxation. The trachea was extubated once spontaneous ventilation of the patient was adequate and the patients were transferred to the PACU. Patient-controlled analgesia (PCA) was applied when the patients opened their eyes in the PACU. PCA consisted of 12 mg of hydromorphone in 100 ml of normal saline and was administered using an AutoMed 3200 pump (AutoMed 3000 Series® Ambulatory Infusion Pump; ACE Medical Corp. Ltd., Seoul, Korea) at a background rate of 1 ml/h and a bolus dose of 1 ml with a lockout interval of 10 minutes. In the PACU and general ward, 1 μg/kg of fentanyl and 50 mg of tramadol were intravenously administered, respectively, as rescue analgesics.

The visual analog scale (VAS) score, amount of PCA administered, rescue analgesics required, and PONV were recorded at the time of discharge from the PACU (T1) and at 2 (T2), 8 (T3), 24 (T4), and 48 hours (T5) after surgery by a designated nurse who was blinded to the group allocation. The nurse was educated on the VAS and PONV by the anesthesiologists. The surgery and anesthesia duration, the first time of eye opening, verbal command response, rescue analgesics requests, and PACU stay duration were also recorded. Patients were discharged from the PACU after achieving a post-anesthesia recovery score (modified Aldrete scale) ≥ 8.

The necessary sample size was calculated based on a pilot study. Seventeen patients in each group were required to detect a difference of “1 over 10” in the VAS score with a power of 0.8 and a type I error of 0.05. To compensate for dropouts and deviations from normality, 40 patients were enrolled. We targeted an 80% probability (β = 0.2) with a significance level (α) of 0.05 and a 10% dropout; thus, 20 patients were required in each group. A statistical analysis was performed using SPSS software (ver. 18.0; SPSS, Inc., Chicago, IL, USA). After assessing normality, continuous data were compared using Student’s *t*-test, while the Mann–Whitney test was performed to compare non-continuous and non-normally distributed data. Chi-squared or Fisher’s exact tests were performed to compare categorical data between the two groups. All data are presented as the mean ± standard deviation. A value of *P* < 0.05 was considered to indicate statistical significance.

## Results

**Figure 1:**
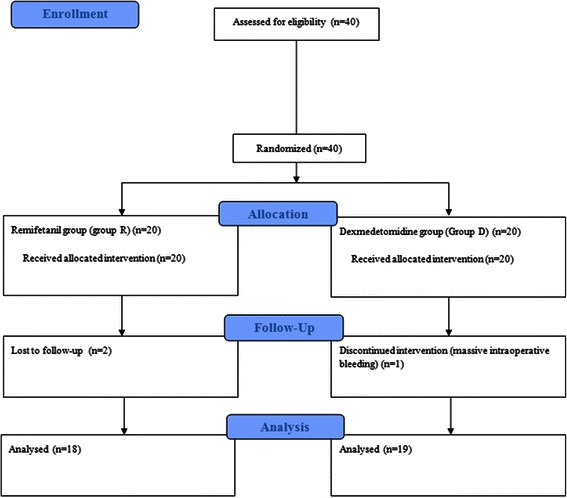
**Consort flow diagram.**

Forty patients were enrolled, of whom two were excluded from Remifentnail group through follow-up loss and one from Dexmedetomidine group because of massive intraoperative bleeding (Figure 1). In total, 37 patients were included in the data analyses and there was no significant difference in demographic characteristics between the two groups (Table 1). The total amount of propofol used was not significantly different. In contrast, the time of eye opening and first verbal command response in the PACU were significantly delayed in Dexmedetomidine group compared to Remifentanil group (*P* < 0.05). In addition, significantly more patients in Remifentanil group required rescue analgesics during the early recovery period in the PACU (*P* < 0.05). However, the PACU stay duration was not significantly different between the two groups (Table 2).

**Table 1:** **Demographic data**

	Remifentanil group	Dexmedetomidine group
Sex (M/F)	8/10	8/11
Age (y)	65.1 ± 5.3	65.9 ± 5.8
Weight (kg)	62.5 ± 6.9	63.1 ± 4.3
Height (cm)	158.0 ± 6.5	159.1 ± 2.1
Hypertension	9 (50)	10 (52.6)
Diabetes mellitus	7 (38.9)	7 (36.8)
Preoperative VAS	3.1 ± 0.7	3.1 ± 1.1

VAS, visual analog scale.

**Table 2:** **Intraoperative and recovery data**

	Remifetnanil group	Dexmedetomidine group	*P*-value
Duration of surgery (min)	171.1 ± 23.2	177.2 ± 23.9	0.376
Duration of anesthesia (min)	212.3 ± 26.3	214.3 ± 21.6	0.314
Propofol used (mg/kg/h)	7.2 ± 1.2	7.8 ± 1.2	0.632
Remifentanil used (μg/kg/min)	0.10 ± 0.03		
Dexmedetomidine used (μg/kg/min)		0.01 ± 0.01	
Time of eye opening (min)	6.9 ± 5.5	21.3 ± 4.9	0.001
Time of first verbal command response (min)	12.8 ± 9.3	23.2 ± 6.8	0.027
Incidence of rescue analgesics requirement, *n* (%)	16 (88.9)	12 (63.2)	0.018
Time of rescue analgesics requirement (min)	13.0 ± 10.2	29.9 ± 11.6	0.011
Incidence of PONV, *n* (%)	5 (27.8)	0 (0)	0.003
Duration of PACU stay (min)	79.2 ± 18.5	76.6 ± 13.5	0.785

Data are presented as mean ± SD or number (proportion).

PONV, postoperative nausea and vomiting; PACU, postanesthesia care unit.

**Figure 2:**
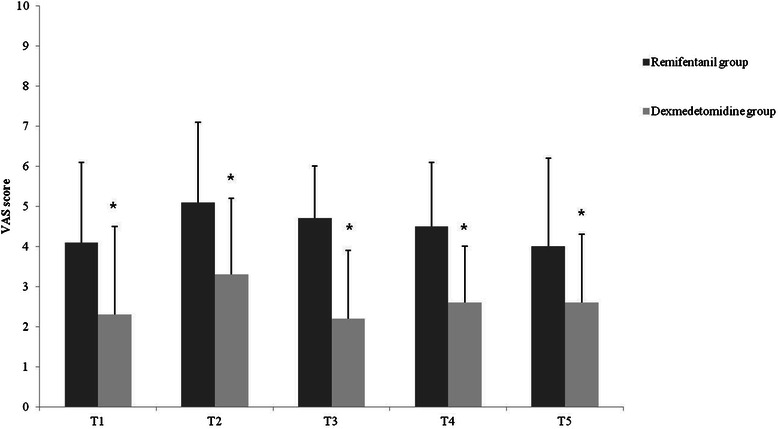
**Comparison of postoperative VAS scores between the groups.** VAS = visual analog scale; T1 = before PACU discharge; T2 = 2 hours after surgery; T3 = 8 hours after surgery; T4 = 24 hours after surgery; T5 = 48 hours after surgery. *P < 0.05.

**Figure 3:**
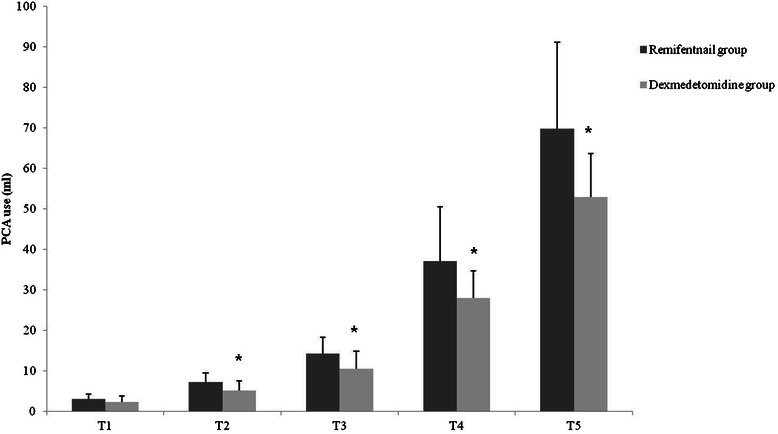
**Comparison of postoperative PCA use between the groups.** PCA = patient-controlled analgesia; T1 = before PACU discharge; T2 = 2 hours after surgery; T3 = 8 hours after surgery; T4 = 24 hours after surgery; T5 = 48 hours after surgery. *P < 0.05.

The VAS score in Remifentanil group was significantly higher than in Dexmedetomidine group at every time point after surgery (4.1 ± 2.0 vs. 2.3 ± 2.2 at T1, and 4.0 ± 2.2 vs. 2.6 ± 1.7 at T5; *P* < 0.05; Figure 2). Dexmedtomidine group had a statistically significantly lower PCA requirement at every time point after surgery except directly before discharge from the PACU (3.0 ± 1.2 ml vs. 2.3 ± 1.4 ml at T1; *P* > 0.05, and 69.7 ± 21.4 ml vs. 52.8 ± 10.8 ml at T5; *P* < 0.05; 1 ml = 0.12 mg hydromophone; Figure 3). Finally, the patients in Remifentanil group required more rescue analgesics at every time point after surgery and displayed more PONV until 24 hours post-surgery (*P* < 0.05; Table 3).

**Table 3:** **Incidence of rescue analgesic requirement and PONV**

	Remifentanil group	Dexmedetomidine group	*P*-value
Incidence of rescue analgesics (*n*)			
Postoperative 2 h (%)	4 (22.2)	0 (0)	0.046
Postoperative 8 h (%)	9 (50)	3 (15.8)	0.038
Postoperative 24 h (%)	10 (55.6)	4 (21.1)	0.045
Postoperative 48 h (%)	9 (50)	3 (15.8)	0.038
Incidence of PONV (*n*)			
Postoperative 2 h (%)	6 (33.3)	0 (0)	0.008
Postoperative 8 h (%)	8 (44.4)	2 (10.5)	0.029
Postoperative 24 h (%)	6 (33.3)	0 (0)	0.008
Postoperative 48 h (%)	2 (11.1)	0 (0)	0.230

Data are presented as number (proportion).

PONV, postoperative nausea and vomiting.

## Discussion

This is the first report in which dexmedetomidine as an adjuvant in propofol-based TIVA has been suggested to alleviate postoperative pain beyond the immediate postoperative period in the PACU. This study demonstrates that dexmedetomidine had superior pain control efficacy compared to remifentanil for the first 48 hours following PLIF surgery, lowering the VAS score and reducing the PCA requirement. Dexmedetomidine also reduced the analgesic requirement and PONV incidence compared to remifentanil. Late postoperative pain may progress to pathological pain, whereas immediate postoperative pain is mainly acute physiological pain; pathologic pain differs from physiologic pain in that it is excessive in intensity and spread and can be activated by low-intensity stimuli and hyperpathia [16]. Therefore, the management of postoperative pain for a longer period in patients undergoing surgeries resulting in severe postoperative pain, including major cancer or orthopedic surgery, is crucial for the long-term postoperative outcome. Unlike previous reports, this study demonstrates that dexmedetomidine is effective for an extended period after surgery, and therefore may improve the postoperative outcome.

Several studies have demonstrated that dexmedetomidine had superior efficacy compared to fentanyl and remifentanil in pain management during a PACU stay [12,13,17]. In these studies, the efficacy of dexmedetomidine in alleviating postoperative pain was focused on the immediate postoperative period; for example, the PACU stay. This may be related to the pharmacokinetics of dexmedetomidine; its elimination half-life is 2–3 hours, with a context-sensitive half-time ranging from 4 to 250 minutes following a 10-minute and an 8-hour infusion, respectively [14]. Although dexmedetomidine has longer action duration than remifentanil, previous investigators may have thought that this would not influence the postoperative period beyond PACU recovery. In contrast, the present study suggests that dexmedetomidine had an effect on pain alleviation until 48 hours after surgery. We attribute this result to the nociceptive cascade. Nociceptors which are located in laminae II-III of the dorsal horn and have a wide dynamic range [18] discharge in proportion to the intensity of stimulation, and high-threshold nociceptors respond only when the stimulus intensity exceeds a threshold. Once nociceptors are sensitized, the threshold for activation is decreased, discharge rate with activation is increased, and rate of basal (spontaneous) discharge is increased, resulting in easier response of nociceptors to incoming stimuli [19]. In addition, postoperative pain itself evokes a higher stress hormone concentration, which in turn produces more intense pain [20]. Dexmedetomidine, with its longer action duration compared to remifentanil, may have reduced the “no pain control period” interval and the time from discontinuation of the adjuvant in propofol-based TIVA to PCA initiation, thereby increasing the stimulus threshold and resulting in a reduced VAS score and PCA requirement.

The present findings were not influenced simply by the longer duration of dexmedetomidine compared to remifentanil. In a previous report, systemic medetomidine alone at subanesthetic did not significantly influence the intensity and thresholds of experimental pain whereas the affective-motivational component of pain was attenuated [21]. The superior efficacy of pain control beyond the known duration of dexmedetomidine (i.e., until 48 hours after surgery) in combination with similar recovery time in PACU suggest that the alleviated postoperative pain over a longer period during recovery might have been influenced by affective-emotional effect of dexmedetomidine rather than the analgesic effect of dexmedetomidine on mechanical stimuli after surgery.

Another reason for the superior postoperative pain control efficacy of dexmedetomidine compared to remifentanil may be related to opioid-induced hyperalgesia (OIH). OIH is characterized by a paradoxical increase in pain intensity or sensitivity in patients receiving opioids at high doses or for an extended duration [22,23]. Numerous studies have suggested that intraoperative remifentanil may paradoxically enhance postoperative pain and hence the opioid analgesic requirement, and this may occur after 60–90 minutes of infusion [24-26]. A recent study demonstrated that intraoperative high-dose remifentanil decreased the mechanical hyperalgesia threshold, enhanced the pain intensity, reduced the time to the first postoperative analgesic requirement, and increased patient morphine consumption, indicating OIH, which was alleviated efficiently using a dexmedetomidine infusion [4]. In the present study, remifentanil was infused over 170 minutes at 0.1 ± 0.03 μg/kg/min, which is sufficient to induce OIH. A higher VAS score and greater PCA requirement imply OIH, although we did not apply any other method to confirm its occurrence.

PONV is one of the most undesirable clinical anesthesia outcomes [27]. Various factors may induce PONV. Perioperative opioid use is a major factor in PONV. In addition, pain itself is an important risk factor for PONV [28,29]. In the present study, dexmedetomidine reduced PONV for 48 hours after surgery, in agreement with a previous study [4]. The greater PCA required may have contributed to the increased PONV incidence in the remifentanil group. More intense pain may have induced PONV and thus made patients require more rescue analgesics, mostly opioid, which in turn aggravated PONV. Therefore, using dexmedetomidine as an adjuvant in propofol-based TIVA may reduce the incidence of PONV by alleviating the pain intensity because of a reduced requirement for postoperative rescue opioids.

## Conclusions

In conclusion, dexmedetomidine as an adjuvant in propofol-based TIVA displayed superior efficacy to remifentanil in alleviating pain and managing postoperative pain for 48 hours following PLIF surgery. It also reduced the requirement for rescue analgesics and PONV. Therefore, dexmedetomidine may be used as an adjuvant in propofol-based TIVA instead of remifentanil for more efficient pain and PONV management.

## Abbreviations

BIS: Bispectral index
OIH: Opioid-induced hyperalgesia
PACU: Post-anesthesia care unit
PCA: Patient-controlled analgesia
PLIF: Posterior lumbar interbody fusion
PONV: Postoperative nausea and vomiting
TIVA: Total intravenous anesthesia
VAS: Visual analog scale
